# An Integrated Imaging Approach to the Study of Oxidative Stress Generation by Mitochondrial Dysfunction in Living Cells

**DOI:** 10.1289/ehp.0901811

**Published:** 2010-04-22

**Authors:** Wan-Yun Cheng, Haiyan Tong, Evan W. Miller, Christopher J. Chang, James Remington, Robert M. Zucker, Philip A. Bromberg, James M. Samet, Thomas P.J. Hofer

**Affiliations:** 1 Department of Environmental Sciences and Engineering, University of North Carolina–Chapel Hill, Chapel Hill, North Carolina, USA; 2 Environmental Public Health Division, National Health and Environmental Effects Research Laboratory, U.S. Environmental Protection Agency, Chapel Hill, North Carolina, USA; 3 Department of Chemistry and the Howard Hughes Medical Institute, University of California–Berkeley, Berkeley, California, USA; 4 Department of Physics, Institute of Molecular Biology, University of Oregon, Eugene, Oregon, USA; 5 Toxicology Assessment Division, National Health and Environmental Effects Research Laboratory, U.S. Environmental Protection Agency, Research Triangle Park, North Carolina, USA; 6 Center for Environmental Medicine and Lung Biology, University of North Carolina–Chapel Hill, Chapel Hill, North Carolina, USA; 7 Helmholtz Zentrum München, German Research Center for Environmental Health, Clinical Cooperation Group Inflammatory Lung Diseases, Gauting, Germany

**Keywords:** biosensors, confocal microscopy, hydrogen peroxide, mitochondrial dysfunction, oxidative stress, real-time imaging, ROS

## Abstract

**Background:**

The mechanisms of action of many environmental agents commonly involve oxidative stress resulting from mitochondrial dysfunction. Zinc is a common environmental metallic contaminant that has been implicated in a variety of oxidant-dependent toxicological responses. Unlike ions of other transition metals such as iron, copper, and vanadium, Zn^2+^ does not generate reactive oxygen species (ROS) through redox cycling.

**Objective:**

To characterize the role of oxidative stress in zinc-induced toxicity.

**Methods:**

We used an integrated imaging approach that employs the hydrogen peroxide (H_2_O_2_)-specific fluorophore Peroxy Green 1 (PG1), the mitochondrial potential sensor 5,5′,6,6′-tetrachloro-1,1′,3,3′-tetraethylbenzimidazolylcarbocyanine iodide (JC-1), and the mitochondria-targeted form of the redox-sensitive genetically encoded fluorophore MTroGFP1 in living cells.

**Results:**

Zinc treatment in the presence of the Zn^2+^ ionophore pyrithione of A431 skin carcinoma cells preloaded with the H_2_O_2_-specific indicator PG1 resulted in a significant increase in H_2_O_2_ production that could be significantly inhibited with the mitochondrial inhibitor carbonyl cyanide 3-chlorophenylhydrazone. Mitochondria were further implicated as the source of zinc-induced H_2_O_2_ formation by the observation that exposure to zinc caused a loss of mitochondrial membrane potential. Using MTroGFP1, we showed that zinc exposure of A431 cells induces a rapid loss of reducing redox potential in mitochondria. We also demonstrated that zinc exposure results in rapid swelling of mitochondria isolated from mouse hearts.

**Conclusion:**

Taken together, these findings show a disruption of mitochondrial integrity, H_2_O_2_ formation, and a shift toward positive redox potential in cells exposed to zinc. These data demonstrate the utility of real-time, live-cell imaging to study the role of oxidative stress in toxicological responses.

Oxidative stress is a common feature of the mechanism of injury induced by a broad range of environmental agents (Finkelstein and Johnston 2004). Such environmental oxidative stress can result directly from the effects of oxidizers, electrophiles, or free radical–generating compounds such as ozone (Steinberg et al. 1990), quinones (Santa-Maria et al. 2005), and redox-active transition metal ions (Valko et al. 2006). Environmental oxidative stress can also involve the depletion of cellular antioxidant defense mechanisms or dysregulation of oxidative metabolism processes in the cell (Ercal et al. 2001).

Analytical methods used to study oxidative stress often focus on the detection of oxidized biomolecules such as oxidized lipids (Kinter 1995), proteins (Kelly and Mudway 2003), and DNA (Aust and Eveleigh 1999). Direct detection of reactive oxygen species (ROS) involved in cellular oxidative stress in living cells has relied heavily on the use of the fluorescent indicator 2,7-dichlorodihydrofluorescein diacetate (H_2_DCF-DA) (Crow 1997). Unfortunately, the interpretation of data obtained with this indicator has been limited by its lack of specificity and by experimental artifacts that include photoinstability, autoxidation, and photoconversion (Marchesi et al. 1999; Rota et al. 1999).

Mitochondria are a known source of partially reduced oxygen species generated as a by-product of oxidative metabolism in the cell (Andreyev et al. 2005). Dysregulation of mitochondrial function with metabolic inhibitors has been shown to induce the release of ROS and associated oxidative stress (Lam et al. 2001). Toxicologically, a wide variety of environmental contaminants ranging from aromatic hydrocarbons (Senft et al. 2002) to heavy metal ions (Valko et al. 2005) have been shown to impair mitochondrial respiration, with ensuing production of ROS. A number of assays measure indices of mitochondrial function, such as ATP concentration, citrate synthase activity, and membrane potential (Ouhabi et al. 1998; Smiley et al. 1991), but the methodologies used to measure mitochondrial redox potential are limited.

In the present study, we used an integrated imaging approach to the investigation of environmental oxidative stress resulting from mitochondrial dysfunction. By applying established and recently introduced indicators, this integrated approach allows real-time measurement of mitochondrial membrane potential, hydrogen peroxide (H_2_O_2_) levels, and redox status in living cells (Cannon and Remington 2008; Rhee 2007). A431 skin carcinoma cells were used as representative of rapidly growing cells with high metabolic rate and energy use that could be inferred to have a correspondingly high mitochondrial activity. Zinc sulfate (ZnSO_4_) was used as a soluble form of the zinc ion (Zn^2+^) that is also environmentally relevant, because sulfate salts are known to predominate among soluble metal salts released by combustion processes. In addition, we used zinc pyrithione, which is used as a mildewcide in outdoor paints, making skin cells a relevant cell type to study with this agent.

We report that exposure to Zn^2+^, a ubiquitous ambient contaminant that has been shown to induce inflammatory (Kim et al. 2006; Tal et al. 2006) and cytotoxic responses (Tang et al. 2001), results in an intracellular accumulation of H_2_O_2_ that is associated with a decrease of mitochondrial reducing redox potential and depolarization of the mitochondrial membrane. These findings demonstrate the utility of an integrated application of imaging techniques for the study of mechanisms of environmental oxidative stress in living cells with improved spatial and temporal resolution as well as specificity.

## Materials and Methods

### Cell culture and experimental settings

A431 human skin carcinoma cells (no. CRL-1555; American Type Culture Collection, Manassas, VA, USA) were used for live cell imaging experiments. The cells were cultured in Dulbecco’s modified Eagle’s medium (DMEM; catalog no. 11995, GIBCO, Grand Island, NY, USA) and supplemented with 10% fetal bovine serum (FBS) and 5 μg/mL gentamicin at 37°C, 5% CO_2_. Cells were seeded on round glass cover slips (22 mm in diameter, thickness #1) in six-well culture plates at 150,000–250,000 cells per well. Cultures were deprived of growth factors overnight prior to study. The A431 cells were preloaded with Peroxy Green 1 (PG1) or 5,5′,6,6′-tetrachloro-1,1′,3,3′-tetraethy-lbenzimidazolylcarbocyanine iodide [JC-1 (T3168; Invitrogen, Carlsbad, CA, USA)], and the cover slip cultures were fitted into a custom-made stainless-steel chamber filled with 500 μL phosphate-buffered saline (PBS), which was supplemented with 1 g/L glucose and kept at 37°C with a stage heater. Conventional and spectral confocal microscopy analyses were conducted using a Nikon Eclipse C1Si confocal microscope (Nikon Instruments Inc., Melville, NY, USA) that was equipped with TE 2000 microscope. Light was delivered to the sample with a 60 × Plan Apo 1.4 numerical aperture (NA) objective; the system also uses diode lasers of 404 nm, 488 nm, 561 nm, and 633 nm. Prior to each experiment, the confocal microscope was tested for field illumination alignment, optical efficiency, colocalization, and axial resolution (Lerner and Zucker 2004; Zucker 2006a, 2006b; Zucker and Lerner 2005; Zucker et al. 2007); and the lens was inspected and cleaned before use. Cells were exposed sequentially to ZnSO_4_ (catalog no. Z-0251; Sigma, St. Louis, MO, USA) at concentrations between 10 μM and 100 μM with or without 4 μM of the Zn^2+^-specific ionophore pyrithione, given at 5 min. The inhibitors apocynin (100 μM) (Miller et al. 2007), wortmannin (10 μM), diphenyleneiodonium (DPI; 25 μM) (Riganti et al. 2004), carbonyl cyanide 3-chlorophenylhydrazone (CCCP; 10 μM) (Bogeski et al. 2006), and Ly294002 (10 μM; all inhibitors were obtained from Sigma), plus compound 56 (C56, 10 μM; Calbiochem, San Diego, CA, USA) (Tal et al. 2006) and EHT 1864 (5 μM; provided by C.J. Der, University of North Carolina–Chapel Hill) (Shutes et al. 2007) were applied 30 min prior to adding Zn^2+^. H_2_O_2_ (1 mM), CCCP (10 μM), or dithiothreitol (DTT; Sigma; 10 mM) was added at the end of experiments as positive controls.

### Measurement of H_2_O_2_

H_2_O_2_ production was monitored using the fluorescein-like Peroxy Green 1 (PG1) dye (Miller et al. 2007). PG1 is a boronate probe with high specificity for H_2_O_2_. A431 cells grown on glass cover slips were labeled in 5 μM PG1 for 15 min at 37°C in PBS glucose solution prior to measurement. PG1 fluorescence was excited using an argon laser (at λ = 488 nm), and the emission spectrum was monitored in a range of λ = 490 nm to 570 nm over 32 channels with 2.5 nm band pass. Signal intensity was quantified at the PG1 emission peak at 523 nm.

### Measurement of mitochondrial membrane potential

The mitochondrial membrane potential was monitored using the fluorescent indicator JC-1. In the presence of physiological mitochondrial membrane potentials, JC-1 forms aggregates that fluoresce with an emission peak at 588 nm. Loss of membrane potential favors the monomeric form of JC-1, which has an emission peak at 530 nm. Cells were labeled with 5 μM JC-1 in DMEM supplemented with 10% FBS and 1 μg/mL gentamicin at 37°C. After 15 min incubation, cells were washed with PBS twice and placed in the chamber with 500 μL PBS glucose. JC-1 fluorescence intensity was monitored with dual excitation at 488 nm and 561 nm and an emission scan range of 490–650 nm (32 channels, 5 nm per channel). Mitochondrial membrane potential was inferred from the ratio of fluorescence intensity of emission maximum at 593 nm and 538 nm, which represented the J-aggregate and monomeric forms, respectively.

### Cardiac mitochondrial swelling assay

Adult pathogen-free female CD-1 mice, which were purchased from Charles River (Raleigh, NC, USA), were used as the source of the cardiac mitochondria. Animals were housed at the U.S. Environmental Protection Agency (EPA) animal care facility (accredited by the Association for Assessment and Accreditation of Laboratory Animal Care) and given *ad libitum* access to both water and food. Animal care was given in accordance with institutional guidelines, and animals were treated humanely, with regard to alleviating suffering. The studies were conducted with approval by the EPA Institutional Animal Care and Use Committee. Mice were euthanized with an intraperitoneal injection of sodium pentobarbital (80 mg/kg body weight), and hearts were excised and weighed. Freshly isolated mitochondria were prepared from the ventricles by differential centrifugation. Briefly, heart tissues were homogenized with three strokes of a polytron homogenizer in ice-cold homogenization buffer containing 225 mM mannitol, 75 mM sucrose, 5 mM morpholinepropanesulfonic acid, and 2 mM taurine, with 0.2% bovine serum albumin (BSA; pH 7.4). The homogenate was transferred to a glass homogenizer and homogenized for five strokes on ice. After centrifugation at 2,500 rpm for 5 min at 40°C, the supernatant was removed and centrifuged at 8,000 rpm for 5 min. The pellet was sequentially washed with homogenization buffer three times and resuspended in homogenization buffer plus 5 mM KH_2_PO_4_. The protein concentration was determined with BSA as a standard by a Bradford assay.

The mitochondria (50 μg) were incubated in buffer containing 120 mM KCl, 10 mM Tris HCl, and 5 mM KH_2_PO_4_ at room temperature. After adding 10 mM glutamate and 2 mM malate, the light scattering of mitochondria was measured at 540 nm for 40 min with a 96-well plate spectrophotometer (POLARstar Optima; BMG, Alexandria, VA, USA). We initiated calcium- or zinc-induced mitochondrial swelling by adding 250 μM calcium or 100 μM zinc and measured for another 20 min. The absorbance was normalized to the initial absorbance.

### Measurement of redox potential in mitochondria

The genetically encoded mitochondria- targeted form of the genetically encoded fluorescent reporter redox-sensitive green fluorescent protein (MTroGFP1) was used for the measurement of redox potential in mitochondria (Hanson et al. 2004). Fugene 6 (catalog no. 11815091001; Roche, Mannheim, Germany) was used for transfection according to the manufacturer’s protocol. The MTroGFP1 plasmid was mixed with Fugene 6 for 30 min at room temperature and applied to the A431 cells for 48 hr. Tetramethyl rhodamine methyl ester (TMRM; catalog no. T-668, Invitrogen), a mitochondria-specific dye, was used to validate the transfection by incubating 500 nM TMRM with transfected cells for 15 min and by visualizing with excitation at 561 nm and with emission filter of 605/75 nm (Chroma Technology Corp, Rockingham, VT, USA). Green fluorescence was derived from excitation at both 404 nm and 488 nm with an emission detected using a band-pass filter of 525/50 nm (Chroma). The results were calculated by rationing the emissions excited by 488 nm and 404 nm laser sequentially.

### Statistical analysis

Imaging data were collected with Nikon EZ-C1 software and quantified by EZ-C1 and Nikon Elements. Figures were plotted with mean ± SE, with three repeat experiments. An average of 5–10 cells with different fluorescence intensities were collected as regions of interests in each experiment and quantified with Nikon EZ-C1 and Nikon Elements software (Nikon Instruments). Pairwise comparisons were carried out using Student’s *t*-test; a *p*-value of < 0.05 was considered statistically significant.

## Results

### Zinc-induced H_2_O_2_ production visualized by PG1 in living cells

**Figure 1:**
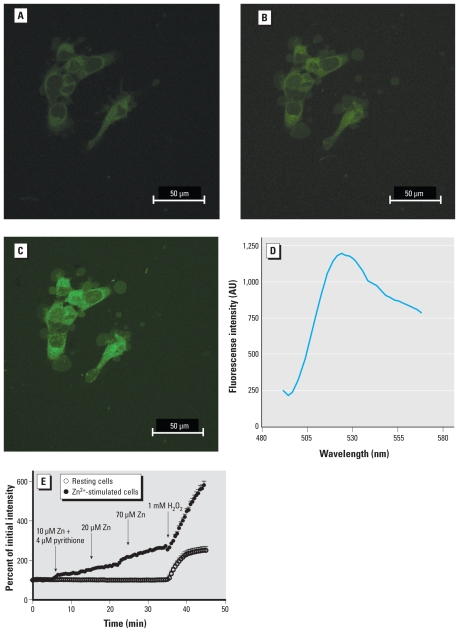
Visualization of zinc-induced H_2_O_2_ production by PG1 fluorescence in A431 cells. A431 cells were incubated with vehicle alone for 5 min (*A*), 100 μM zinc sulfate for 30 min (*B*), or 1 mM H_2_O_2_ given at 45 min (*C*). (*D*) Emission spectra confirmation of PG1 fluorescence with peak at 523 nm; intensity is shown in arbitrary units (AU). (*E*) Time course of H_2_O_2_ production detected by PG1 fluorescence in resting cells and in cells stimulated with 10 μM Zn plus 4 μM pyrithione at 5 min, 20 μM Zn at 15 min, or 70 μM Zn at 25 min; H_2_O_2_ (1 mM) was added at 35 min as a positive control for both experimental conditions. Triplicate observations were made for control and stimulated cells with an average of 10 cells in each run. Data are mean ± SE.

As a model toxicant for these studies, we used Zn^2+^, a non-redox-active metal that is ubiquitously found associated with particulate matter in ambient air and occupational settings. Addition of noncytotoxic concentrations (Tal et al. 2006) of Zn^2+^ and pyrithione (10–100 μM ZnSO_4_ plus 4 μM pyrithione) to A431 cells resulted in a time-dependent elevation in intracellular concentrations of H_2_O_2_ as detected by an increase in PG1 fluorescence intensity (Figure 1A,B). H_2_O_2_ added as positive control resulted in a marked increase (550%) in PG1 fluorescence (Figure 1C). Spectral analysis of PG1 fluorescence excited with 488 nm revealed an emission peak at 523 nm (Figure 1D), consistent with published reports (Miller et al. 2007). Sequential images were captured at 30-sec intervals and plotted as the relative fluorescence intensity normalized to initial intensity. Cells exposed to 100 μM Zn^2+^ for 10 min showed a 64% increase in PG1 fluorescence intensity relative to starting levels. PG1-loaded resting cells not exposed to Zn^2+^ observed during the same testing period showed < 4% increase in fluorescence intensity (Figure 1E). Similar to resting cells, A431 cells incubated in 4 μM pyrithione alone did not show an increase in PG1 fluorescence intensity (data not shown).

### Identification of the source of Zn^2+^-induced H_2_O_2_

As shown in Figure 1, Zn^2+^-stimulated H_2_O_2_ production was visualized as an increase in fluorescence disseminated throughout the cytosol. To identify the intracellular source of H_2_O_2_, cells were pretreated with the nicotinamide adenine dinucleotide phosphate oxidase inhibitors apocynin or DPI, the epidermal growth factor receptor kinase activity inhibitor C56, the phosphoinositide 3-kinase activity inhibitors wortmannin or Ly294002, the Rac GTPase kinase inhibitor EHT 1864, or the mitochondrial uncoupler CCCP, prior to exposure to Zn^2+^ (Bogeski et al. 2006; Riganti et al. 2004; Shutes et al. 2007; Vanhaesebroeck et al. 2001). With the exception of CCCP, the application of these inhibitors did not have statistically significant effects on Zn^2+^-induced H_2_O_2_ production in A431 cells (Table 1). Pretreatment with CCCP induced a statistically significant 32% inhibition in PG1 fluorescence intensity relative to Zn^2+^ alone. Treatment with other reagents resulted in less than 10–20% inhibition of the H_2_O_2_-dependent PG1 fluorescence production induced by Zn^2+^ (Table 1). These findings implicate mitochondria as the source of Zn^2+^-induced H_2_O_2_ production.

### Zinc-induced mitochondrial dysfunction

**Figure 2:**
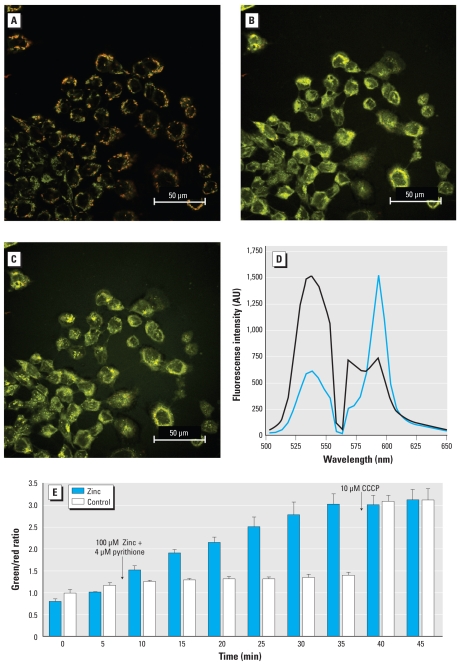
Measurement of mitochondrial membrane potential visualized by JC-1 in A431 cells treated with zinc. A431 cells treated with vehicle alone (*A*) or with 100 μM zinc before (*B*) and after addition of 10 μM CCCP (*C*) as a positive control. (*D*) The spectrum of JC-1 is shown under 2 different conditions; control cells (blue line) and depolarized cells (black line). Intensity is shown in arbitrary units (AU). (*E*) Measurement of JC-1 fluorescence intensity (taken as the ratio of green to red) in control and Zn^2+^-exposed A431 cells; 100 μM zinc plus 4 μM pyrithione were added at 5 min, and 10 μM CCCP was added to both groups at 35 min. Images were obtained with simultaneous excitation of 488 nm and 561 nm laser and emission scan range between 490 nm and 650 nm using a 5 nm band pass. Triplicate observations were made for control and stimulated cells with an average of 10 cells in each run. Data are mean ± SE.

The maintenance of the electron transport chain proton gradient by functional mitochondria establishes a transmembrane electrical potential that can be monitored using the fluorescence indicator JC-1. Intrinsically a green indicator with an emission maximum at 529 nm in monomeric form, JC-1 accumulates in functional mitochondria in concentrations sufficient to form J-aggregates, which leads to a shift of the emission maximum to 588 nm (Figure 2A). Zinc-induced mitochondrial depolarization led to a change in the equilibrium of JC-1 observed as a shift of the JC-1 emission maximum to a shorter wavelength (Figure 2B), corresponding to an emission peak shift from 538 nm to 588 nm (Figure 2D). The ratio of the fluorescence emission at 538 and 588 nm represents the degree of Zn^2+^-induced mitochondrial depolarization (Guthrie and Welch 2008). Loss of mitochondrial membrane potential was observed 10 min after cells were exposed to 100 μM Zn^2+^ and 4 μM pyrithione, and continued to rise for 30 min (Figure 2E). CCCP was added at the end of each experiment as a positive control. As shown in Figure 2E, the addition of CCCP did not induce a further increase in fluorescent intensity in cells exposed to Zn^2+^, suggesting a complete depolarization of mitochondrial potential induced by Zn^2+^ in A431 cells.

**Figure 3:**
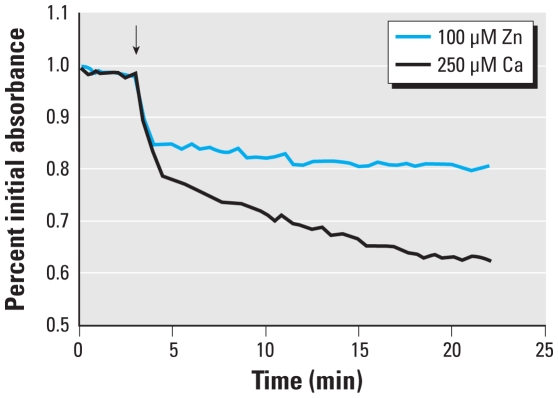
Zinc-induced mitochondrial dysfunction measured using the swelling assay. A suspension of isolated cardiac mitochondria was monitored for absorbance at 550 nm after the addition of 100 μM Zn^2+^ or 250 μM calcium ion. Absorbance values were normalized to the initial reading. Data represent three independent experiments.

As an independent measurement of mitochondrial function, we next examined the effect of Zn^2+^ exposure on the mitochondrial membrane transition pore using the mitochondrial swelling assay in isolated cardiac mouse mitochondria. This particular assay requires a large number of isolated mitochondria that would be impractical to obtain from cultured cells. Therefore, mouse heart mitochondria were used for this purpose as a model suitable for toxicological testing. Treatment with Zn^2+^ and pyrithione resulted in significant swelling of isolated mitochondria. The addition of calcium (positive control) induced spontaneous swelling, indicated by a 17% decrease in absorbance at 4 min, whereas the addition of zinc induced a similar effect, with a 15% decrease in absorbance (Figure 3). These results independently confirmed that Zn^2+^ directly affects mitochondrial function.

### Visualization of Zn^2+^-induced oxidative stress in mitochondria

**Figure 4:**
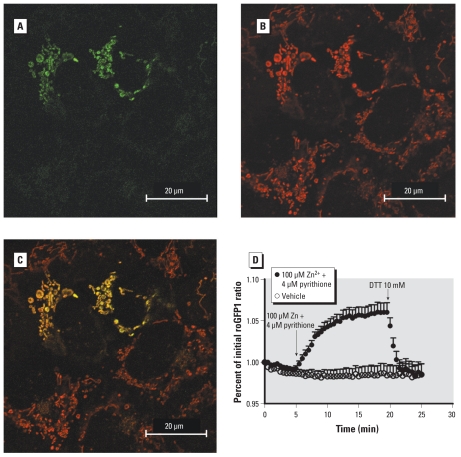
Zinc-induced oxidative stress in mitochondria. (*A*) A431 cells transfected with MTroGFP1 demonstrate green fluorescence associated with mitochondria. (*B*) Cells incubated with the mitochondrial indicator TMRM, shown as red fluorescence. (*C*) Colocalization of the two images. (*D*) Mitochondrial redox potential was monitored as the ratio of fluorescence intensity under 404/488 excitation normalized to the value at 0 min; vehicle or 100 μM Zn^2+^ plus 4 μM pyrithione was added at 5 min. DTT (10 mM) was added at 20 min as a positive control. Data were grouped from 20 cells studied over three separate experiments, expressed as mean ± SE.

The data presented above indicate that the mitochondrion is a target of Zn^2+^-mediated toxicity and a potential source of ROS and oxidative stress. To examine the effect of Zn^2+^ exposure on mitochondrial redox potential, we used MTroGFP1 (Hanson et al. 2004). This genetically encoded reporter responds to changes in redox status with changes in the relative intensity of fluorescence at 510 nm upon excitation with its two excitation maxima, 404 and 488 nm. Cells transfected with MTroGFP1 displayed the expected green fluorescence in a pattern that was exclusively associated with mitochondria (Figure 4A). Confirming the localization of the sensor, the MTroGFP1 fluorescence colocalized with a validated mitochondrial indicator, TMRM (Figure 4B,C). Exposure of these cells to 100 μM Zn^2+^ and 4 μM pyrithione induced a rapid increase in the ratio of fluorescence at 404/488, indicating a less reduced redox (Figure 4D). This Zn^2+^-induced change corresponded to a loss of mitochondrial reducing potential, from a value previously reported to be − 288 mV (Hanson et al. 2004), toward a more positive redox potential, starting within 2 min and reaching a plateau by 10 min (Figure 4D). Subsequent addition of 10 mM DTT as a positive control restored a negative mitochondrial redox potential.

## Discussion

Oxidative stress is increasingly recognized as an important feature of the mechanism of toxic action that is common to many structurally disparate environmental contaminants (Valko et al. 2005). However, a significant limitation in the investigation of oxidative stress in toxicology has been the availability of an integrated methodological approach for real-time detection of reactive oxidant species and oxidative damage in living cells. Compared with conventional biochemical assays, live-cell imaging offers superior temporal and spatial resolution of intracellular processes. In the present study, we have employed an integrated imaging approach to study oxidative stress associated with mitochondrial dysfunction in cells exposed to the environmental air pollutant Zn^2+^. The detection of specific ROS is an important feature of this approach. The indicator H_2_DCF-DA and its variants have been used widely for ROS detection by imaging of living cells. However, several limitations are associated with H_2_DCF-DA, including a lack of ROS specificity and its susceptibility to oxidation by different species such as peroxynitrite, nitric oxide, superoxide, and H_2_O_2_ under various experimental conditions (Crow 1997). In addition, H_2_DCF-DA can also be oxidized by heme and hemoproteins (Ohashi et al. 2002) and is subject to photoreduction (Marchesi et al. 1999), further reducing the reliability of ROS detection with this indicator.

Using PG1, we visualized Zn^2+^-stimulated H_2_O_2_ production in living cells. Although PG1 is not specifically targeted to an intracellular compartment, when used in combination with classical inhibitors, as we did in this study, it can be used to infer an intracellular source of H_2_O_2_ within the cell. PG1 consists of a fluorescein-like dye conjugated to a chemoselective boronate switch that responds to H_2_O_2_ with high specificity (Miller et al. 2007). PG1 is the first fluorescence-based molecular indicator for the specific detection of H_2_O_2_ with sufficient sensitivity to detect low concentrations of peroxide such as those produced by nonphagocytes responding to physiological signals (Miller et al. 2007). Thus, as an ROS sensor, PG1 represents a significant improvement over H_2_DCF-DA in that it offers superior specificity, sensitivity, and stability (Rhee 2007). Miller et al. (2007) showed that PG1 has high specificity for H_2_O_2_ relative to a wide range of other oxygen, nitrogen, chlorine, and organic oxidant species. Lending credence to these findings, in the present study we showed that Zn^2+^, a transition metal incapable of producing ROS by redox cycling, induces H_2_O_2_ production detected by PG1 fluorescence, which we independently show to be the result of mitochondrial dysfunction induced by Zn^2+^ exposure.

A critical element of the approach applied in this study is monitoring redox potential using redox-sensitive variants of green fluorescent protein (roGFPs). These genetically encoded reporters were first described by Hanson et al. (2004) as redox potential sensors with two fluorescence excitation maxima, thus permitting ratiometric analysis that minimizes errors associated with variations in indicator concentration (roGFP expression), illumination intensity, and cell thickness. The roGFP sensors were genetically engineered to respond to changes in intracellular thiol-disulfide equilibria (Hanson et al. 2004) and therefore provide a noninvasive method for measuring cellular redox potential. The roGFP sensors feature fast response rates and selectivity for midpoint potential and for subcellular compartment targeting (Cannon and Remington 2006; Dooley et al. 2004; Lohman and Remington 2008). They have been used for monitoring redox status in plant cells and ischemic neuronal cells under various conditions (Schwarzlander et al. 2009; Vesce et al. 2005). Here, we used a mitochondria-targeted version, MTroGFP1, to monitor the effect of Zn^2+^-induced oxidative stress. The reporter responded as expected to treatment with exogenous oxidants (H_2_O_2_) and reducing agents (DTT) within 2 min. Our findings show that Zn^2+^ induces a rapid decrease of mitochondrial reducing potential, consistent with the independent measurements of reduced mitochondrial membrane potential and opening of the mitochondrial permeability pore.

Previous studies point to Zn^2+^-induced inhibition of mitochondrial respiration by binding to the bc1 complex (Link and von Jagow 1995; Lohman and Remington 2008). Furthermore, mitochondrial energy metabolism is known to be inhibited through α-ketoglutarate dehydrogenase complexation by Zn^2+^ (Brown et al. 2000). In addition, it has been shown that treatment with soluble Zn^2+^ has resulted in substantial respiration block, mitochondrial structural alterations along with mitochondrial permeability transition changes, and ROS production in isolated mitochondria (Bossy-Wetzel et al. 2004). Exposure to Zn^2+^ is also associated with dysregulation of signaling leading to increased expression of inflammatory mediators (Kim et al. 2006; Tal et al. 2006), as well as apoptotic (Franklin and Costello 2009) and necrotic cell death (Tang et al. 2001). Based on current understanding, the impairment of mitochondrial function observed in this study can be seen as an underlying mechanism through which the oxidant responses are caused by Zn^2+^ exposure, or interpreted to represent a secondary effect of the oxidant stress induced by Zn^2+^. Additional studies will be required to elucidate the complex mechanisms that underlie the oxidative stress associated with Zn^2+^ toxicity.

As our findings with Zn^2+^ in this study demonstrate, by integrating the MTroGFP redox sensor and the JC-1 sensor of mitochondrial membrane potential with conventional methods to determine mitochondrial function (the swelling assay and metabolic inhibitors), it is possible to elucidate the mechanism of oxidative stress induced by exposure to environmental agents. The conclusion that oxidative stress induced by Zn^2+^ originates in the mitochondria is supported by the following findings in the present study: *a*) the partial inhibition of Zn^2+^-stimulated H_2_O_2_ production in the cytosol by the mitochondrial respiration uncoupler CCCP, *b*) the observation that Zn^2+^ exposure induces mitochondrial depolarization, *c*) the finding that Zn^2+^ induces mitochondrial swelling (in isolated cardiac mitochondria), and *d*) the decrease of reducing redox potential induced by Zn^2+^ exposure.

The methodologies used in this study are broadly applicable to other oxidative stressors such as redox active transition metals and organic oxidants. Using BEAS 2B cells as a model of the human airway epithelium, we have observed that the diesel exhaust organic constituents 1,2-napthoquinone and *p*-benzoquinone induce H_2_O_2_ production and a change in redox potential in both cytosolic and mitochondrial compartments (Cheng WY et al., unpublished observations).

The timeline of events measured with the integrated approach using real-time imaging of living cells in this study seems to support a sequence in which exposure to Zn^2+^ results in mitochondrial dysfunction, possibly an arrest of electron transport (Link and von Jagow 1995; Lorusso et al. 1991), causing an accumulation and release of partially reduced oxygen species, which gives rise to the increase in cytoplasmic H_2_O_2_ detected as increased PG1 fluorescence subsequent to mitochondrial depolarization and redox changes.

The change in redox potential detected by the MTroGFP1 sensor appears to precede Zn^2+^-induced depolarization of mitochondria reported by the color shift in JC-1 fluorescence. However, it is likely that the relative timing of the changes in fluorescence reported by the sensors and indicators used in this study is also influenced by differences between their individual response rates. Thus, it is not currently possible to precisely establish the sequence of events based on changes in the fluorescence intensity of different sensors. Future studies will focus on refining this integrated approach to factor in response rates in a dynamic system and thus more accurately reflect the temporal sequence of cellular events involving mitochondrial dysfunction and ROS generation leading to oxidative stress induced by ambient toxicants.

## Figures and Tables

**Table 1:** The effect of inhibitors on Zn^2+^-induced H_2_O_2_ production.

Inhibitor^a^	Concentration (μM)	Percent inhibition
Apocynin	100	21
DPI	25	7
C56	10	3
Wortmannin	10	10
Ly294002	10	0
EHT 1864	5	5
CCCP	10	32*

a Cells incubated with inhibitors in various concentrations 30 min prior to 100 μM Zn^2+^ exposure. Inhibition effects were calculated by comparison of Zn^2+^-induced PG1 fluorescence after 30 min exposure with or without prior inhibitor treatment.

* Denotes statistically significant difference from vehicle control, *p* < 0.05.
